# Amelogenesis Imperfecta with Anterior Open Bite: A Rare Case Report

**DOI:** 10.5005/jp-journals-10005-1118

**Published:** 2011-04-15

**Authors:** Ruchi Singhal, Anuradha Pathak, Puneet Goenka

**Affiliations:** 1 Demonstrator, Department of Pedodontics and Preventive Dentistry, Government Dental College, Rohtak, Haryana, India; 2 Professor and Head, Department of Pedodontics and Preventive Dentistry, Government Dental College, Patiala, Punjab, India; 3 Senior Lecturer, Department of Pediatric and Preventive Dentistry, Mahatma Gandhi Dental College, Jaipur, Rajasthan, India

**Keywords:** Amelogenesis imperfecta, Open bite, Composite laminate veneers, Stainless steel crowns.

## Abstract

This clinical report describes the treatment plan for a young patient affected by amelogenesis imperfecta with anterior open bite. The objectives of the treatment were to eliminate tooth sensitivity while enhancing esthetics and restoring masticatory function. Treatment included resin composite laminate veneers on maxillary anterior teeth and stainless steel crowns for posterior teeth.

## INTRODUCTION

Amelogenesis imperfecta (AI) has been described as a group of hereditary conditions that disturbs the developing enamel structure and exists independent of any related systemic disorder.^1-3^ This enamel anomaly affects both the primary and permanent dentition.^2^ The incidence of AI has been reported to vary between approximately 1:700 and 1:16,000, depending on the population studied and the diagnostic criteria used.^2^

There are three types of AI: Hypoplasia, hypocalcifica-tion and hypomaturation. In the hypoplastic forms, the enamel does not develop to its normal thickness. In the hypocalcified forms, the enamel thickness on the newly erupted teeth closely approaches that of normal teeth, but the enamel is soft, friable and can easily be removed from the dentin. Hypomaturation is an abnormal occurrence in the final stages of the mineralization process. Hypomatu-ration differs from hypocalcification in that the enamel is harder, but with a mottled opaque white to yellow-brown or red-brown color.^2^

According to Seow, the primary clinical problems of AI are esthetics, dental sensitivity and decreased occlusal vertical dimensions.^4^ Restoration of these defects is important not only because of esthetic and functional concerns, but also because there may be a positive psychological impact for the patient.^1^ Treatment planning for patients with AI is related to many factors: Age, socioeconomic status of the patient, the type and severity of the disorder and the intraoral situation at the time the treatment is planned.^1,4^

An interdisciplinary approach is necessary to evaluate, diagnose and resolve esthetic problems using a combination of orthodontic, prosthodontic and restorative treatment.

This clinical report describes the treatment of a 14-year-old boy with AI using resin composite laminate veneers in the anterior region and stainless steel crowns in the posterior region.

## CASE REPORT

**Fig. 1:**
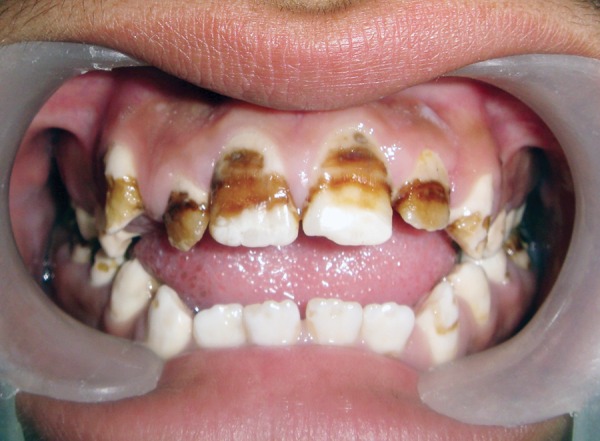
Pretreatment view of teeth in maximum intercuspation (note: the anterior open bite)

**Fig. 2:**
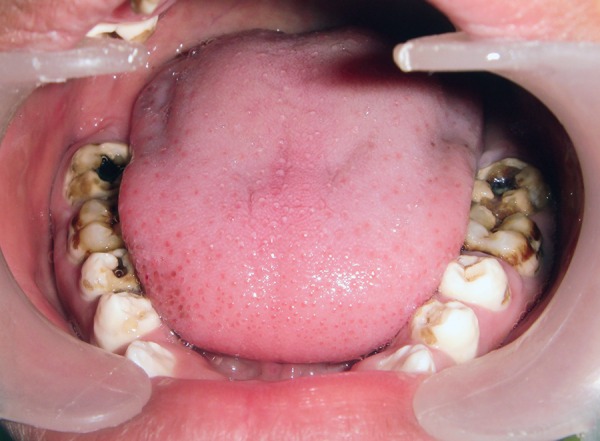
Pretreatment mandibular occlusal view

A 14-year-old boy reported to the Pediatric Dental Clinic with the chief complaint of discolored anterior teeth since they erupted. Patient also complained of sensitivity while taking cold food in the posterior teeth of lower jaw on both the sides and in the maxillary incisors. The medical and dental history was noncontributory. There was a history of similar discoloration of teeth in the mother of patient. Photographs and dental radiographs were made. Diagnostic casts were prepared. Clinical examination of patient revealed anterior open bite deformity (Fig. 1). All the molars were carious and showing attrition (Fig. 2). Maxillary an-teriors show pit defects and yellowish brown discoloration (Fig. 1). The exposed dentin was hypersensitive. After thorough examination, the patient was diagnosed as having hypomaturation type of AI because the thickness of enamel was normal and hard in texture but mottled, opaque white to yellow brown in color.

A treatment plan was developed with the following objectives: To improve the esthetics, to reduce the reported sensitivity of the teeth and to restore the masticatory function. Restoration of anterior teeth with composite laminate veneers and posterior teeth with stainless steel crowns was planned. The patient and the parents were informed of the diagnosis and the treatment plan, which they accepted.

**Fig. 3:**
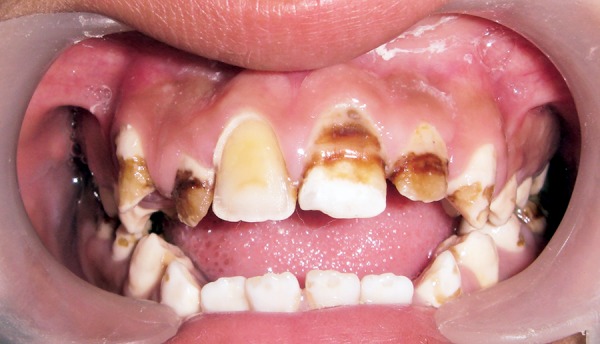
Facial reduction of maxillary incisor for composite laminate veneering

**Fig. 4:**
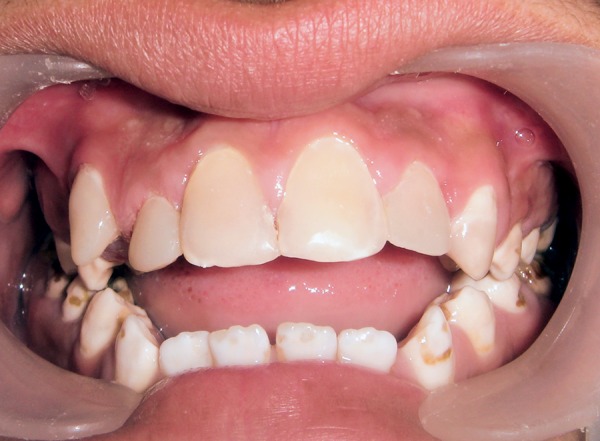
Posttreatment view of teeth in maximum intercuspation

A 0.5 mm facial reduction was done on the maxillary anterior teeth for resin composite laminate veneers (Fig. 3). Care was taken to confine the tooth preparation within the enamel to facilitate better bonding of composite to the tooth. The prepared surfaces were acid etched and restored with an adhesive system (Prime & Bond NT) and resin composite (Esthet-X, Dentsply, York, PA, USA) (Fig. 4).

**Fig. 5:**
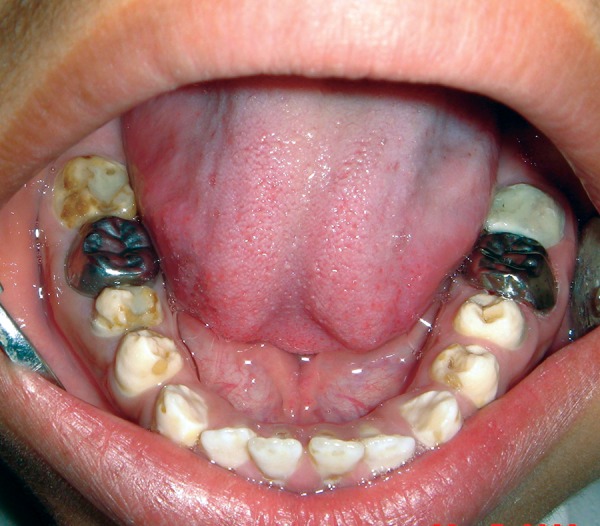
Posttreatment mandibular occlusal view

The mandibular first permanent molars were prepared to receive stainless steel crowns. Preformed stainless steel crowns (3M ESPE, St Paul, Minn) were fitted on the molars without any endodontic treatment to stabilize the occlusion and to halt attrition (Fig. 5). Minor proximal reduction was undertaken for proper fit of the crowns. Crowns were cemented using luting Glass Ionomer Cement (GC Fuji I, GC Corporation, Tokyo, Japan). Occlusal relationship was reevaluated. The second permanent molars were restored with composite resin (Esthet-X, Dentsply, York, PA, USA). Patient was kept on regular recall appointments.

## DISCUSSION

The common conditions that affect enamel structure other than AI include congenital syphilis, fluorosis and vitamin (A, C and D) deficiency. No significant medical history along with associated hereditary pattern in our patient helps us in establishing our diagnosis.

The rehabilitation of AI in a young patient must take into account the development of the child’s teeth, their pulpal morphology and the skeletal jaw growth. The goal of the treatment is to establish an esthetic appearance and an efficient masticatory function until adulthood.

AI is frequently found in association with anterior open bite (AOB).^5,6^ The incidence of AOB in patients with AI varies from 24-60%.^5^ Weinmann et al (1945) suggested that AOB is the result of defects in the eruptive mechanism, secondary to the disturbances of the enamel epithelium.^5^ Witkop and Sauk (1976) suggested that tongue interposition, probably provoked by an increased sensitivity of the teeth to hot and cold, resulted in mechanical interference, i.e. the alveolar growth is impeded by tongue interpositioning.^5,7^ Rowley et al (1982), however, reported that AOB was associated with vertical hyperplasia characterized by a large gonial angle, large maxillary-mandibular plane angles and increased facial height.^5^ They suggested that the frequent association of AOB and AI is caused by a genetically determined anomaly of craniofacial development, rather than by local factors influencing alveolar growth. The treatment of AOB in patient with AI is more complicated and more challenging. Presurgical orthodontics is often not feasible because of lack of crown height and condition of enamel. A multisegment Lefort I intrusion osteotomy with or without bilateral sagittal split osteotomy, whether or not followed by prosthetic rehabilitation is usually the treatment of choice.^6^ In the present case, surgery was delayed until jaw growth was completed to prevent postsurgical discrepancy. Other common causes of AOB include tongue thrusting and thumb sucking habit. In the present case, no such habit has been associated with the patient.

There are number of alternatives for treatment of anterior teeth affected by AI which includes full coverage coronal restorations (crowns), porcelain laminate veneers and resin composite laminate veneers. Full coverage crowns and porcelain laminate veneers require removal of substantial amounts of tooth structure, thus more invasive and include risk of pulp exposure in young permanent teeth. Similarly for posterior teeth, metal-ceramic or porcelain-fused metal crowns are commonly used. But the relatively high pulp horns in young patients lead to pulp exposure during tooth preparation. The preferred alternative in such cases is preformed stainless steel crowns, which require least tooth preparation.

**What this paper or case report adds?**

- This paper is a brief review of etiology, types and clinical manifestations of different types of AI
- It describes the clinical evaluation and common problems faced by the patients affected by this condition
- It also describes the frequent association of AI with anterior open bite, which is often misdiagnosed as separate entity and is not correlated with AI.

**Why this paper or case report is important to pediatric dentists?**

- This case report adds a new dimension to the management of young patients affected with AI. Majority of the case reports described in literature included adult patients, whose management differs from that of a young patient.^1,2,4,8^
- The rehabilitation of AI in a young patient must take into account the development of the child’s teeth, the health of the periodontal tissues and the mandibular and maxillary growth.
- It is essential to monitor the occlusion closely over several months and to conserve pulp vitality in immature permanent teeth so they can complete their growth cycle.
